# Ferromagnetism in sphalerite and wurtzite CdS nanostructures

**DOI:** 10.1186/1556-276X-8-17

**Published:** 2013-01-07

**Authors:** Zhaolong Yang, Daqiang Gao, Zhonghua Zhu, Jing Zhang, Zhenhua Shi, Zhipeng Zhang, Desheng Xue

**Affiliations:** 1 Key Laboratory for Magnetism and Magnetic Materials of MOE, Lanzhou University, Lanzhou, 730000, People’s Republic of China

**Keywords:** CdS nanostructures, Hydrothermal methods, Room-temperature ferromagnetism, Sulfur vacancies

## Abstract

Room-temperature ferromagnetism is observed in undoped sphalerite and wurtzite CdS nanostructures which are synthesized by hydrothermal methods. Scanning electron microscopy and transmission electron microscopy results indicate that the sphalerite CdS samples show a spherical-like shape and the wurtzite CdS ones show a flower-like shape, both of which are aggregated by lots of smaller particles. The impurity of the samples has been ruled out by the results of X-ray diffraction, selected-area electron diffraction, and X-ray photoelectron spectroscopy. Magnetization measurements indicate that all the samples exhibit room-temperature ferromagnetism and the saturation magnetization decreases with the increased crystal sizes, revealing that the observed ferromagnetism is defect-related, which is also confirmed by the post-annealing processes. This finding in CdS should be the focus of future electronic and spintronic devices.

## Background

Since the first discovery of ferromagnetism (FM) in Mn-doped GaAs [1], great effort has been paid to search for intrinsic dilute magnetic semiconductors (DMSs) with Curie temperatures (*T*_c_) at or above room temperature (RT) by doping semiconductors with transition metals (TMs) [2,3]. During the past few years, room-temperature ferromagnetism (RTFM) has been reported in TM-doped DMSs experimentally. Nevertheless, the mechanism of the observed FM remains controversial theoretically, which mainly includes experimental artifacts, segregation of secondary ferromagnetic phases, magnetic clusters, and indirect exchange mediated by carriers, electrons, and holes associated with impurities that are related to the observed RTFM [4-7]. Subsequently, RTFM has also been observed in undoped semiconducting or insulating (such as HfO_2_, In_2_O_3_, MgO, ZnO, SnO_2_, etc.) [8-12], where nominal magnetic ions are not present, and the term ‘*d*^*0*^ FM’ [13,14] was suggested to summarize these cases. It is strongly believed that the point defects in semiconductors or insulators have an open-shell electronic configuration, which can indeed confine the compensating charges in molecular orbitals, forming a local magnetic moment. Recently, experiment results show that the size of the lower dimensional systems, such as film thickness or diameter of nanoparticles, has an effect on the vacancy concentration as well as their magnetic behavior [15,16]. The results are also supported by theoretical works which show the effects of curvature, confinement, and size on various properties of nanocrystals [17,18]. Obviously, the surface-to-volume atomic ratio will be increased significantly with the decreased size of nanocrystals. Since the surface has a broken atomic symmetry and it often has higher anisotropy, new surface states that differ from their bulk form are established, which play a crucial role in controlling the electronic, optical, and other properties of nanocrystals.

CdS, belonging to the II-VI compound family, has a considerably important application such as in optoelectronic devices, photocatalysts, solar cells, optical detectors, and nonlinear optical materials [19-25]. If RTFM were achieved in CdS, it would be a potential candidate in the fabrication of new-generation magneto-optical and spintronic devices. Remarkably, lots of investigations have demonstrated FM with *T*_c_ above room temperature observed in transition metal ion (such as Fe, Co, Cr, Mn, and V)-doped CdS-based low-dimensional materials [26-30]. Recently, Pan et al. demonstrated that FM can be realized in CdS with C doping via substitution of S which can be attributed to the hole-mediated double-exchange interaction [18]. Li et al. also studied a Cu-doped CdS system by first-principles simulation and predicted that the system shows a half-metallic ferromagnetic character and the *T*_c_ of the ground state is above RT [31]. Meanwhile, Ren et al. indicated that Pd doping in CdS may lead to a long-range ferromagnetic coupling order, which results from *pd* exchange coupling interaction [32]. Moreover, Ma et al. studied the magnetic properties of non-transition metal/element (Be, B, C, N, O, and F)-doped CdS and explained the magnetic coupling by *pp* interaction involving holes [33]. In this paper, we report the observation of size-dependent RTFM in CdS nanostructures (NSs). The CdS NSs in sphalerite and wurtzite structures were synthesized by hydrothermal methods with different sulfur sources. The structure and magnetic properties of the samples were studied.

## Methods

CdS NSs were synthesized by hydrothermal methods. In a typical procedure for the synthesis of sphalerite CdS samples, 0.15 M cadmium chloride (CdCl_2_ · 2.5H_2_O) and 0.15 M sodium thiosulfate (Na_2_S_2_O_3_ · 5H_2_O) were added into 40 mL deionized water. After stirring for 30 min, the mixed solution was transferred into a Teflon-lined stainless steel autoclave of 50-mL capacity. After being sealed, the solution was maintained at 90°C for 2, 4, 6, and 8 h, which were denoted as S1, S2, S3, and S4, respectively. The resulting solution was filtered to obtain the samples. To eliminate the impurity ions, the products were further washed with deionized water for several times and then dried in air at 60°C. Wurtzite CdS were synthesized with different sulfur sources. In this method, 0.2 M cadmium chloride (CdCl_2_ · 2.5H_2_O) and 0.2 M thioacetamide (CH_3_CSNH_2_) were added into 40 mL deionized water. After stirring, the cloudy solution was transferred into a Teflon-lined stainless steel autoclave of 50-mL capacity. After being sealed, the solution was maintained at 60°C for 4, 6, 8, and 10 h, which were denoted as S5, S6, S7, and S8, respectively. The as-formed wurtzite CdS NSs were filtered, washed with deionized water, and then dried in air at 40°C.

X-ray diffraction (XRD; X’Pert PRO PHILIPS with Cu K_α_ radiation, Almelo, The Netherlands) was employed to study the structure of the samples. The morphologies of the samples were obtained using a scanning electron microscope (SEM; Hitachi S-4800, Chiyoda-ku, Japan). Microstructures of the samples were characterized using a transmission electron microscope (TEM; Tecnai TMG2F30, FEI, Hillsboro, OR, USA) and high-resolution TEM (HRTEM) equipped with selected-area electron diffraction (SAED) and energy-dispersive X-ray spectrum (EDS). The measurements of static magnetic properties were made using a Quantum Design MPMS magnetometer based on a superconducting quantum interference device (SQUID; San Diego, CA, USA). Electron spin resonance (ESR; JEOL, JES-FA300, microwave frequency is 8.984 GHz, Akishima-shi, Japan) spectra were recorded to study the dynamic magnetic properties of the samples. The chemical bonding state and the compositions of the samples were determined by X-ray photoelectron spectroscopy (XPS; VG Scientific ESCALAB-210 spectrometer, East Grinstead, UK) with monochromatic Mg K_α_ X-rays (1,253.6 eV). The thermogravimetric and differential thermal analysis (TG-DTA; DuPont Instruments 1090B, Parkersburg, VA, USA) was employed to obtain the variation of mass and phase transition details of the samples during argon annealing.

## Results and discussion

**Figure 1:**
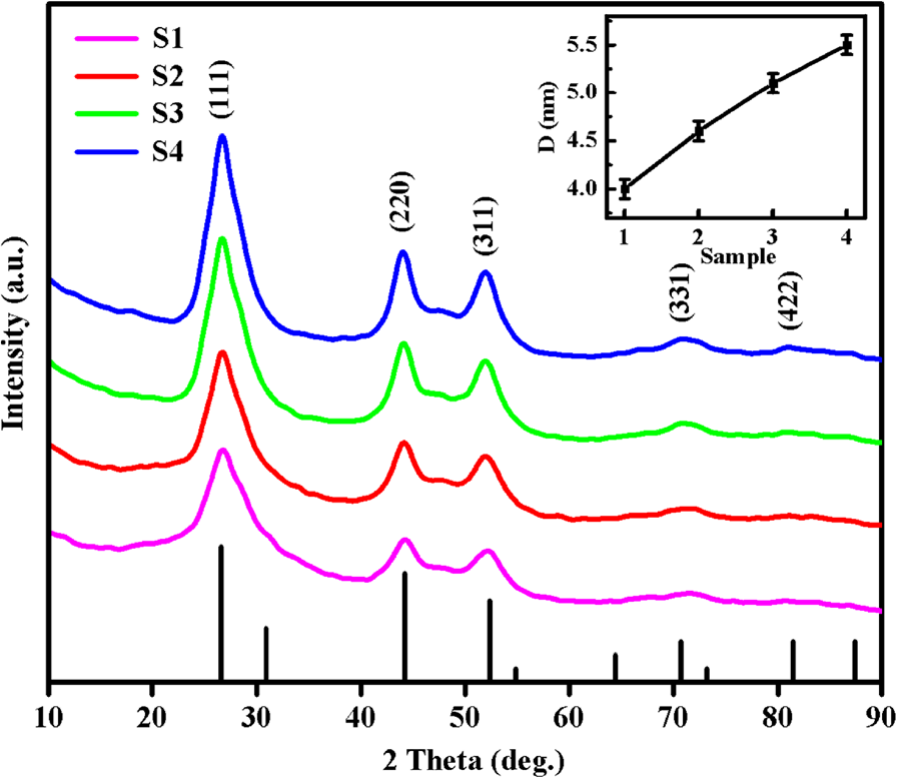
**XRD patterns of samples S1 to S4 represented by lines of different colors.** The inset shows average crystal size of samples S1 to S4 calculated by the Scherrer formula.

**Figure 2:**
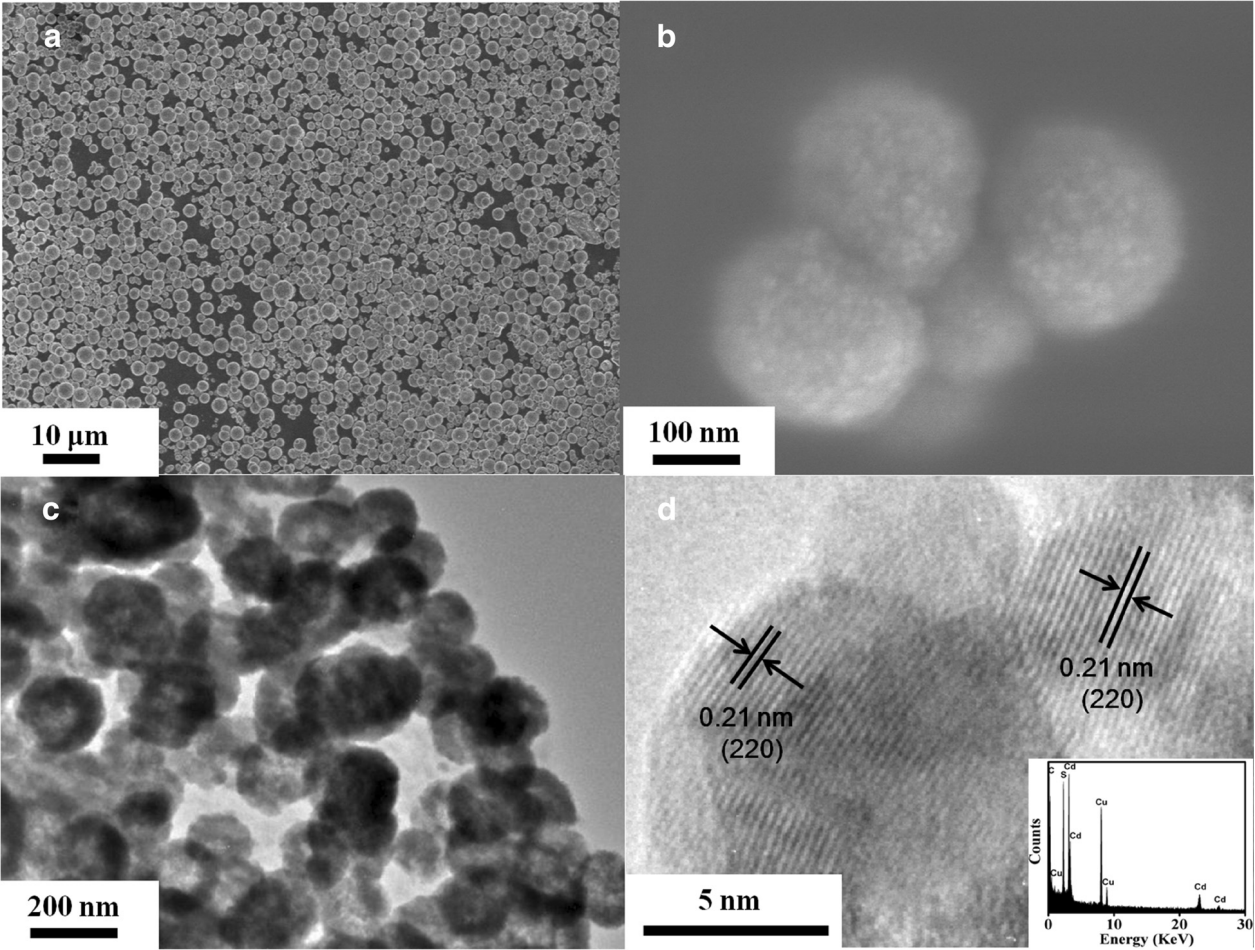
**Morphological characteristics of sphalerite CdS NSs.** (**a**) SEM image of sample S1. (**b**) SEM image of representative spherical particles in sample S1. (**c**) TEM image and (**d**) HRTEM image of sample S1. The inset shows corresponding EDS result.

Structural analysis of sphalerite CdS NSs synthesized at different times (samples S1 to S4) was carried out by XRD, and the results are shown in Figure 1. All diffraction peaks can be indexed to the cubic sphalerite structure of CdS (JCPDS card no. 10–0454). The absence of any other peaks suggests that there is no secondary phase present. Using the Scherrer formula for the full width at half maximum of the main peaks, the average crystalline size has been estimated to be around 4.0, 4.6, 5.1, and 5.5 ± 0.1 nm for samples S1 to S4 (inset of Figure 1), which implies the increase of the crystalline size as the synthesis time increases. Figure 2a,b shows the SEM images of sample S1. Clearly, all products are in the form of a spherical particle with diameters around 200 nm. Under high magnification, it obviously shows that each spherical particle is made up of smaller parts. Figure 2c shows the TEM image of sample S1; it reveals that many crystalline grains congregate together to form a spherical particle and the average size is about 200 nm, which matches the SEM result. It can be clearly seen from the HRTEM of sample S1 in Figure 2d that a single-crystalline grain is about 4 nm in diameter, which is consistent with the XRD result, and it has a lattice spacing of 0.21 nm equaling to the interplanar spacing of the sphalerite CdS in (220) plane. The EDS result is shown in the inset of Figure 2d. The result shows that only the elements Cd, S, C, and Cu are present; Cd and S have an atomic ratio of 54:46. C and Cu are from the carbon membranes which hold the samples during measurement.

**Figure 3:**
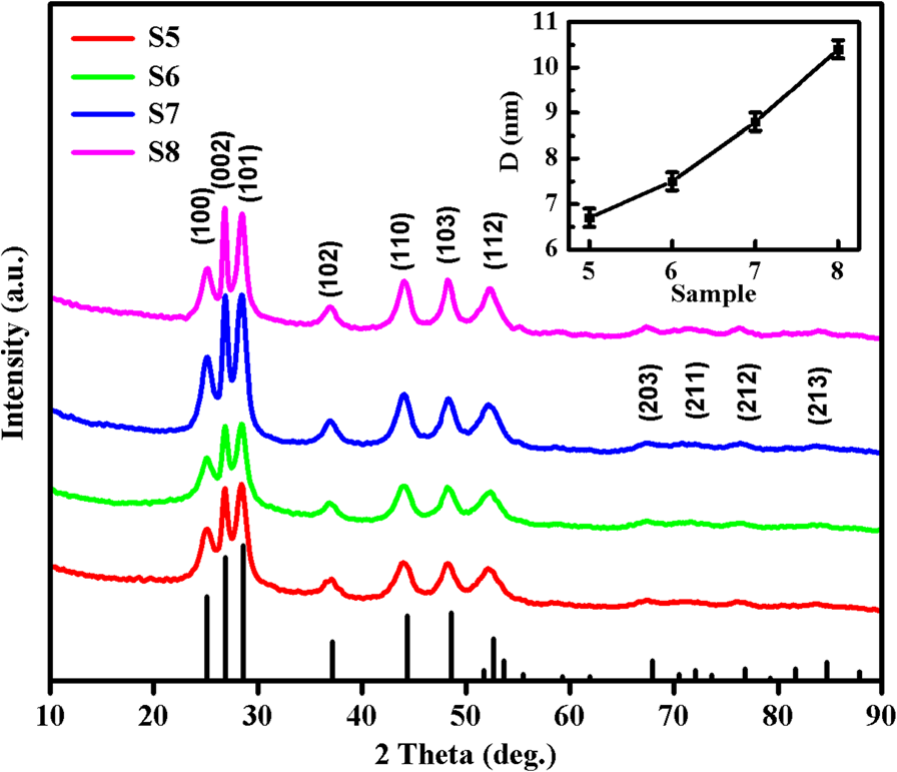
**XRD patterns of samples S5 to S8 represented by lines of different colors.** The inset shows average crystal size of samples S5 to S8 calculated by the Scherrer formula.

**Figure 4:**
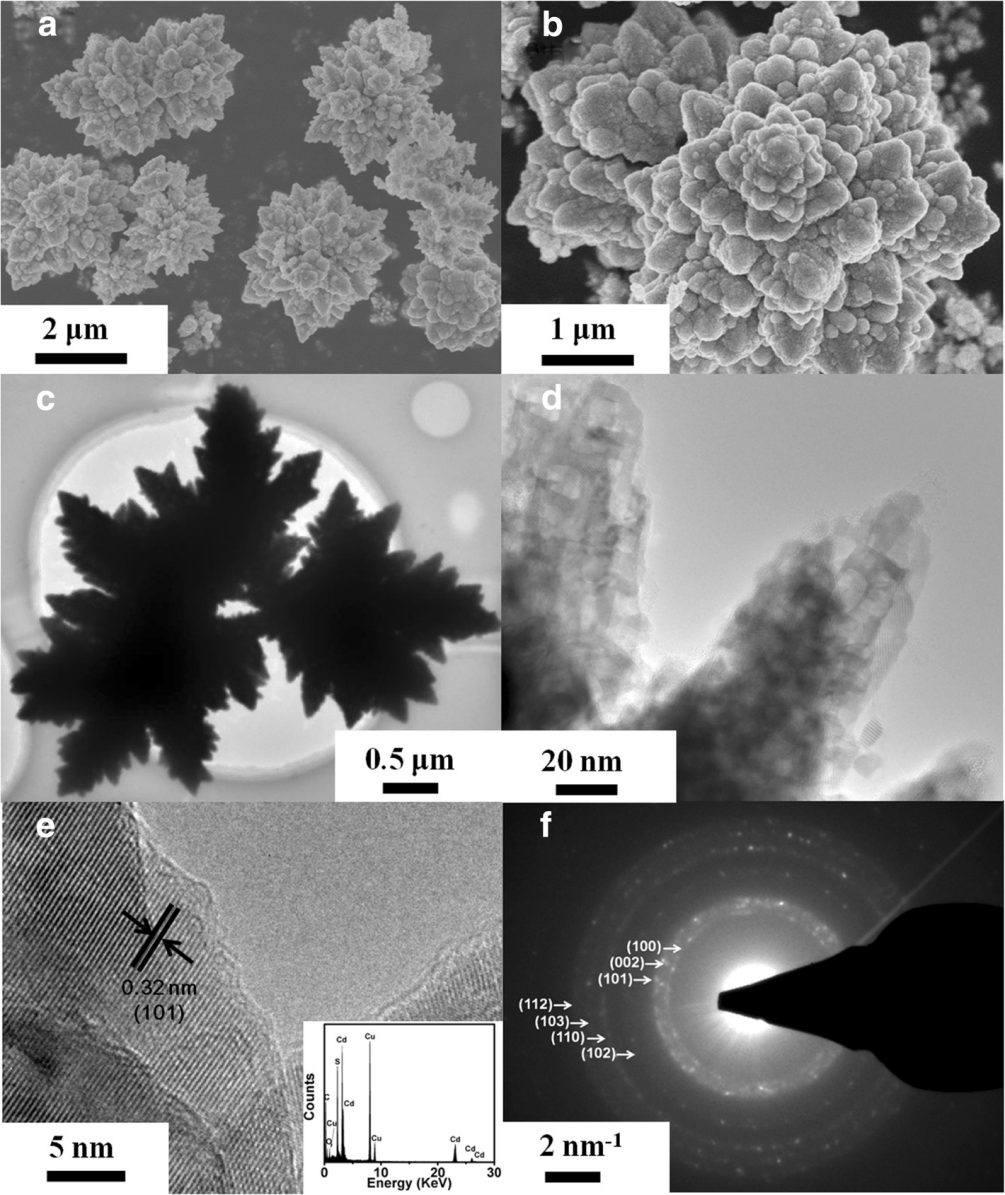
**Morphological characteristics of wurtzite CdS NSs.** (**a**, **b**) SEM images of the flower-shaped wurtzite CdS nanostructures (S5). (**c**, **d**) TEM images of sample S5. (**e**) HRTEM and EDS (inset) results for the same sample (S5). (**f**) The corresponding SAED pattern.

Figure 3 displays the XRD patterns of samples S5 to S8, which confirm the formation of a single hexagonal wurtzite structure without impurity phase (JCPDS card no. 41–1049). Size-dependent XRD broadening is also observed in these samples, implying the decrease of the average crystal size as the synthesis time decreases. Figure 4a,b shows the SEM image of sample S5, revealing that the particles aggregate into a flower shape spontaneously. The TEM images in Figure 4c,d show the shadow of the flower-shaped nanostructures which matches the SEM results above. The subsequent HRTEM image shown in Figure 4e confirms the formation of well-crystalline particles, and the lattice spacing is 0.32 nm, which is equal to the lattice constant of the standard wurtzite CdS in (101) plane. The EDX result shows that only Cd and S are present in the sample (inset of Figure 4e). Figure 4f depicts the result of corresponding SAED, and all the diffraction rings were indexed to the wurtzite phase of CdS, where the agreement with the XRD pattern is excellent.

**Figure 5:**
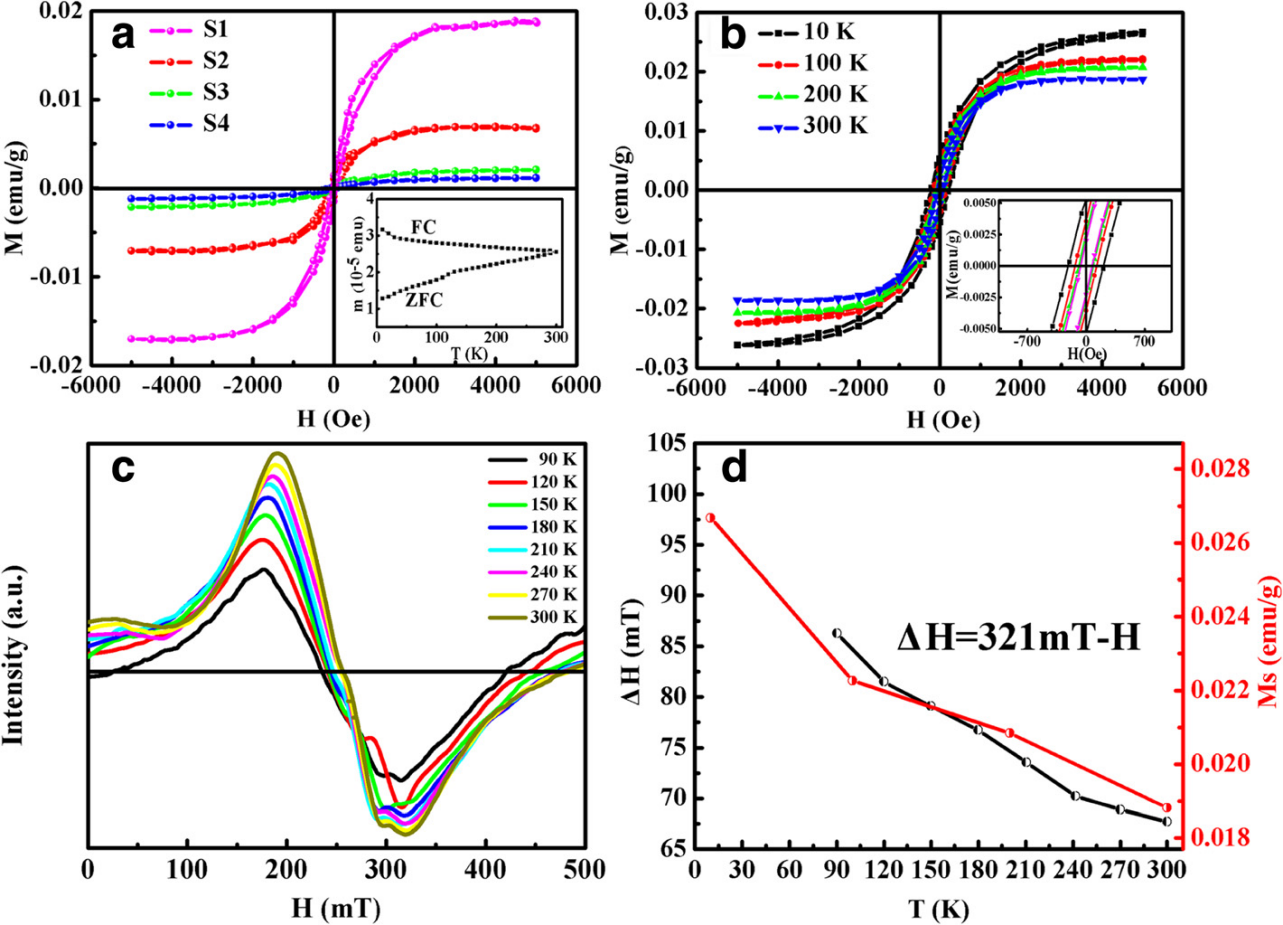
**Magnetic characteristics of sphalerite CdS NSs represented by lines of different colors.** (**a**) Room-temperature *M*-*H* curves of samples S1 to S4. The inset shows ZFC and FC curves with a dc field of 100 Oe applied on sample S1. (**b**) *M*-*H* curves for sample S1 measured at different temperatures. (**c**) ESR spectra of sample S1 measured from 90 to 300 K. (**d**) The calculated Δ*H* which is *H*_center_ is far from 321 mT (*g* = 2.0023) and the variation of *M*_s_ at different temperatures for the same sample (S1).

The magnetization versus magnetic field (*MH*) curves for samples S1 to S4 are displayed in Figure 5a which were measured at 300 K under the maximum applied magnetic field of 5,000 Oe using a sample holder of high-purity capsules free from any metallic impurity. The same measurement procedures were done for the empty capsule, which shows that it is diamagnetic, and the diamagnetic signal of the capsule was subtracted from the measured magnetic signal of the samples. The hysteresis loops suggest that all samples exhibit clearly RTFM. It is worth noticing that the saturation magnetization (*M*_s_) strongly depends on the crystalline size of samples: *M*_s_ decreases from 0.0187 to 0.0012 emu/g with the increasing crystalline size from 4.0 to 5.5 nm. The *d*^*0*^ ferromagnetism in undoped oxide and sulfide nanoscale materials are often considered as the result of crystal defects [13,14,34]. It is to be sure that the defect grows mostly in the boundary and surface of the crystal grain. Because the volume fraction of the interface could be rather small, the ferromagnetic parts should be small either [35]. The inset of Figure 5a shows zero-field-cooled (ZFC) and field-cooled (FC) magnetization curves of sample S1 in the temperature range of 10 to 300 K at 100 Oe. The dividable curve reveals that the *T*_c_ of the sample is above 300 K. Furthermore, there is no blocking temperature in this temperature range, indicating that the observed RTFM is an intrinsic attribute rather than caused by ferromagnetic impurities [36,37]. The *MH* curves for sample S1 measured at different temperatures from 10 to 300 K are shown in Figure 5b. The diamagnetic signal due to the sample holder was subtracted, and the magnetization was saturated at about 3,000 Oe. It can be seen that the *M*_s_ decreases with the increasing temperature. What’s more, the sample shows considerable hysteresis, and the coercive field decreases in a monotonic fashion from a value of 210 Oe at 10 K to 69 Oe at 300 K, which is a typical ferromagnetic behavior.

ESR was performed to further characterize the magnetic properties of the sphalerite CdS NSs. Figure 5c depicts the ESR results measured at different temperatures from 90 to 300 K for sample S1. It can be seen that the sample shows resonance signals with applied magnetic field from 0 to 500 mT. The center magnetic fields (*H*_center_) for the sample are far from 321 mT which characterize a free electron (*g* = 2.0023), indicating that the sample has obvious FM [38], and the ferromagnetic coupling between the moments increase with the decreasing temperature. According to the theory of ferromagnetic resonance [38], the relationship between resonance field and microwave frequency in the ferromagnetic resonance is *hν* = *gμ*_B_ · *H*, where *h*, *ν*, *g*, *μ*_B_, and *H* are the Planck constant, frequency of the applied microwave magnetic field, *g*-factor, Bohr magnetron, and resonance magnetic field, respectively. In FM materials, the orbital angular momentum quenching in the crystal field and *g*-factor is 2.0023; the resonance field is made up of applied field *H*_a_ and magnetocrystalline anisotropy field *H*_k_: *H* = *H*_a_ + *H*_k_. If we define *H*_a_ as *H* and attribute the change of *H*_k_ to the *g*-factor, which is defined as an effective *g*-factor (*g*_eff_), then the ferromagnetic resonance relationship changes to *hν* = *g*_eff_*μ*_B_ · *H*_a_. *H*_k_ will increase with the decreasing temperature, and then *g*_eff_ will get higher. In sample S1, the *g*_eff_ increases from 2.54 to 2.74 as the temperatures decrease from RT to 90 K. The results in Figure 5d indicate that the variation of Δ*H* (=321 mT − *H*_center_, which represents the position of the resonance peak) measured at different temperatures is consistent with that of *M*_s_ in the samples.

**Figure 6:**
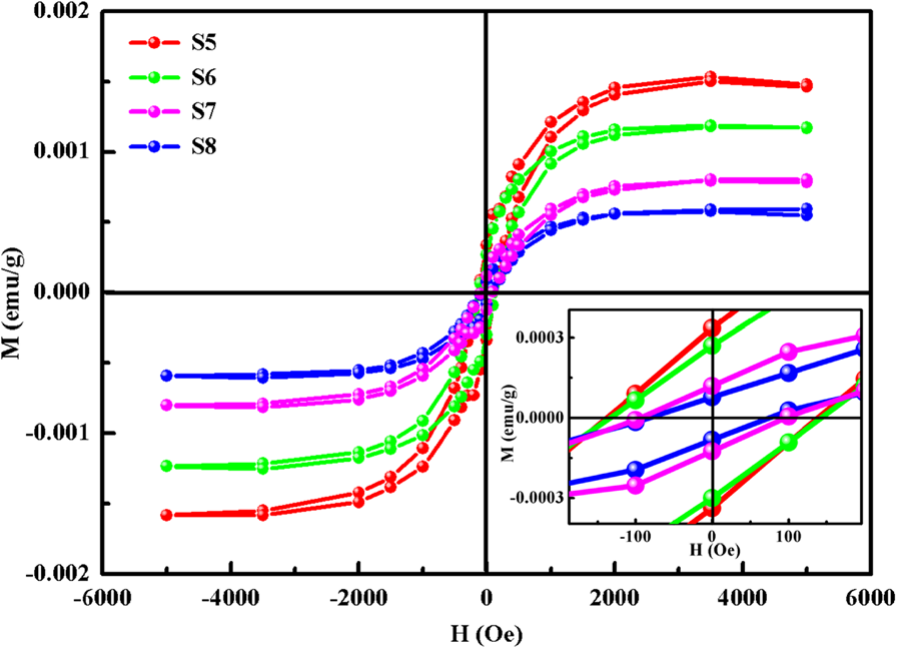
***M*-*H *curves of wurtzite CdS NSs represented by lines of different colors.***M*-*H* curves of samples S5 to S8 measured at RT; the inset shows a magnified view of the low-field data.

As mentioned above, wurtzite CdS NSs were prepared by a hydrothermal method using a different sulfur source. The *M*-*H* curves measured at room temperature for samples S5 to S8 are shown in Figure 6, where the diamagnetic signal has been subtracted. Results indicate that all samples also exhibit clear hysteresis loops; the smaller crystal size shows the largest *M*_s_ (about 0.0015 emu/g), and with increasing crystal size, the *M*_s_ decreases. The variation of *M*_s_ is similar to that of sphalerite CdS.

**Figure 7:**
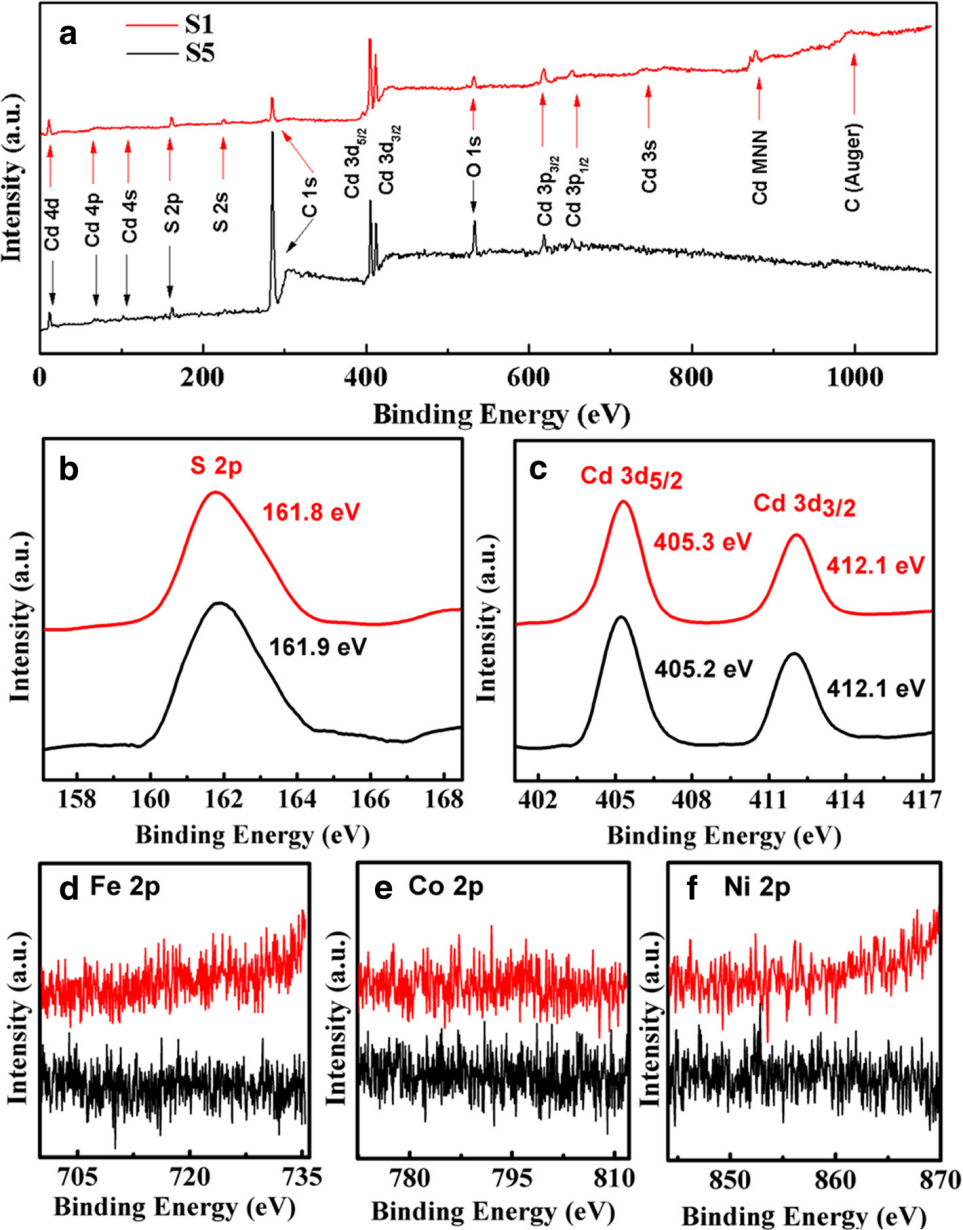
**XPS spectra represented by lines of different colors.** (**a**) XPS survey spectra, high-resolution scan of S 2*p* (**b**) and Cd 3*d* (**c**) of samples S1 and S5. Absence of magnetic elements Fe, Co, and Ni has been confirmed by the core-level XPS spectra of Fe 2*p* (**d**), Co 2*p* (**e**), and Ni 2*p* (**f**).

The composition and purity of the CdS NSs were obtained by XPS. Representative spectra of the sphalerite-structure CdS NSs (sample S1) and wurtzite-structure CdS NSs (sample S5) are shown in Figure 7a. The results show that only the elements Cd, S, C, and O are present, where the standard C 1*s* peak at 284.6 eV was used as a reference for correcting the shifts and O is from O_2_ adsorbed on the sample. The S 2*p* and Cd 3*d* core-level binding energy spectra are shown in Figure 7b,c, respectively. For the Cd 3*d* spectra, peaks correspond to the core level of 3*d*_5/2_ and 3*d*_3/2_ at 405.3 eV (405.2 eV for sample S5) and 412.1 eV, and for the S 2*p* spectra, the core level of 2*p* is at 161.8 eV (161.9 eV for sample S5), corresponding to previous reports [39]. Calculation of relative chemical compositions for S1 shows that Cd and S have an atomic ratio of 57.3:42.7, which demonstrates the existence of high density of sulfur vacancies, and this result is consistent with that of EDS. More importantly, the core-level XPS spectra of Fe 2*p*, Co 2*p*, and Ni 2*p* (Figure 7d,e,f) confirm that there is no magnetic impurity present in the sample. Therefore, it can be concluded that the observed FM in all CdS samples is intrinsic and caused by sulfur vacancies.

**Figure 8:**
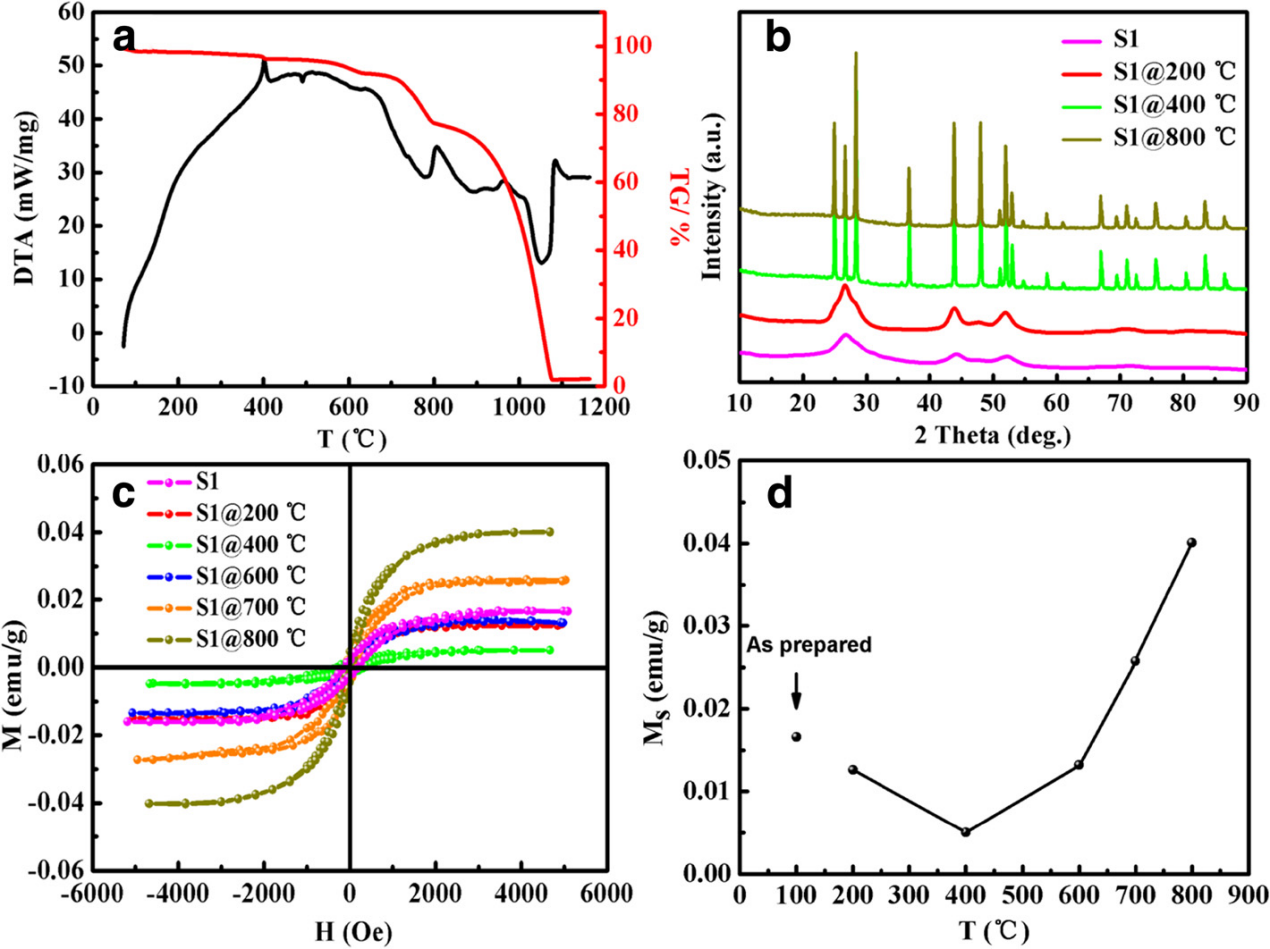
**Post-annealing results represented by lines of different colors.** (**a**) DTA-TG curve for sample S1 which was performed in Ar atmosphere from 60°C to 1,200°C. (**b**) The representative XRD patterns for sample S1 annealed at 200°C, 400°C, and 800°C. (**c**) *M*-*H* curves of the post-annealing samples. (**d**) Variation of *M*_s_ for sample S1 after post-annealing processes.

Magnetic properties of the post-annealing samples further confirmed the defect-related FM in CdS samples. To obtain the annealing details, the TG and DTA were measured for sample S1, in which the test was performed in argon atmosphere with a heating rate of 60°C/min. As shown in Figure 8a, the DTA for sample S1 indicates that there is a phase transition from sphalerite to wurtzite between 300°C and 400°C which corresponds to the sharp exothermic peak in the DTA curve, and this result is further confirmed by XRD [40]. Above 900°C, an endothermic peak occurs in the DTA curve and the mass decreases radically which is shown in the TG curve. These results indicate that the CdS sample begins to decompose at 900°C and vanishes completely above 1,100°C (where the mass becomes 0% in the thermogravimetric curve). In follow-up experiments, sample S1 was divided into several parts and placed in ceramic boats, then annealed in argon with a gas flow rate of 40 sccm. The post-annealing temperature was kept at 200°C, 400°C, 600°C, 700°C, and 800°C. The temperature was kept constant for 120 min and then cooled naturally in argon. XRD results for the post-annealing samples shown in Figure 8b indicate that the sample annealed at 200°C still shows the sphalerite phase, but the wurtzite structure appeared when the annealing temperature increased. It can also be seen that when the annealing temperature exceeds 400°C, the phase structure of the samples transforms to wurtzite completely and undergoes fine crystallization.

The *MH* curves for the post-annealing samples and the variation of their *M*_s_ are shown in Figure 8c,d, respectively. It can be seen that the *M*_s_ of the samples decrease continuously after post-annealing at 200°C and 400°C. However, the *M*_s_ increases with the increasing annealing temperature when the annealing temperature exceeds 400°C. The chemical composition calculated from the XPS result shows that Cd and S have an atomic ratio of 76.7:23.3 for sample S1 after being annealed at 800°C, which indicates that the density of sulfur vacancies gets higher than that of the as-prepared sample. As the analysis of the above annealing progresses, it can be understood that argon annealing at a temperature lower than 400°C results in crystal grain reconstruction and growth which compensates the sulfur vacancies. However, when the annealing temperature gets higher, the sample begins to decompose and promotes large amount of vacancies. Subsequently, the exchange interaction between these different concentrations of sulfur vacancies changes the *M*_s_. Note that changes of *M*_s_ for the wurtzite-structure samples after post-annealing processes have the same variation as those for the sphalerite ones above. The post-annealing results further clarify the role of sulfur vacancies in triggering the RTFM in undoped CdS [34,41].

## Conclusions

In summary, well-crystalline CdS NSs both in sphalerite and wurtzite were synthesized by simple hydrothermal methods. The NSs were self-aggregated into spherical and flower shapes, respectively. Intrinsic FM is observed in the samples by the magnetic hysteresis loops and prominent ferromagnetic resonance signals. The mechanism of RTFM from sulfur vacancies is proposed. Moreover, the magnetization value can be tuned by changing the concentration of sulfur vacancies, which is affected by the particle size and annealing condition. These findings not only demonstrate that pure CdS shows tunable RTFM, but also suggest that introduction of sulfur vacancies can be a significant way to mediate the *d*^*0*^ FM.

## Competing interests

The authors declare that they have no competing interests.

## Authors’ contributions

ZY prepared all the samples, participated in all of the measurements and data analysis, and drafted the manuscript. DG and DX conceived and designed the manuscript. ZZ1 carried out the XPS measurements and data analysis. JZ participated in the SQUID and TG-DTA measurements. ZZ2 carried out the XRD measurements and data analysis. ZS participated in the data analysis and interpretation of the results. All authors have been involved in revising the manuscript and read and approved the final manuscript.
